# Impact of Coronavirus Disease Lockdown on Children With Type 1 Diabetes Mellitus in Al-Khobar, Saudi Arabia

**DOI:** 10.7759/cureus.21350

**Published:** 2022-01-17

**Authors:** Asma A Alsalman, Mai R Aldossari, Zahra D Alomani, Sukainah I Alkhunaizi, Zahrah A Aljardah, Fatima A Almousa, Zahra A Kalalah

**Affiliations:** 1 Medicine, Imam Abdulrahman Bin Faisal University, Dammam, SAU; 2 Family and Community Medicine, King Fahad University Hospital, Dammam, SAU

**Keywords:** saudi arabia., al-khobar, pediatrics, covid-19 lockdown, type 1 diabetes

## Abstract

Background

After the emergence of the novel coronavirus disease pandemic, Saudi Arabia implemented lockdown restrictions to counter this infection and prevent its spread. There have been a lot of challenges to the majority of people, especially diabetic patients, which might result in an increased risk of developing complications and significant lifestyle changes, including physical inactivity, unhealthy diet, and psychological distress.

Objective

This study aims to assess the impact of coronavirus disease lockdown on children with type 1 diabetes (T1D) mellitus in Al-Khobar, Saudi Arabia.

Methods

This is a questionnaire-based cross-sectional study which was conducted in pediatric patients, aged ≤18 years old, with type I diabetes, who were attending the endocrine clinic at the King Fahd Hospital of the University (KFHU) in Al-Khobar, Saudi Arabia. A total of 250 patients were identified from the hospital databases, a total of 164 patients were included in the study, and one of the parents/guardians was interviewed through mobile phone after taking their consent.

Results

The medication compliance during the lockdown has significantly increased as the percentage of children who did not miss any dose of medications increased to 92.7% in comparison to 70.7% before the lockdown (P=0.000). Also, diabetic ketoacidosis (DKA)-related intensive care unit admission had significantly decreased during the lockdown (P=0.001). Regarding the number of consumed meals, it increased from 53.7% up to 68.3% before and during the lockdown period, respectively (P=0.005). In addition, there was no significant change in children's psychological health during the lockdown period (p= 0.123).

Conclusion

The study indicates that there was an increase in compliance to medications, a significant decrease in intensive care unit and emergency department admission due to hypo/hyperglycemia during the lockdown period. Medical practitioners are encouraged to educate diabetic patients regarding the importance of self-blood glucose monitoring, healthy lifestyle, psychological wellbeing, and regular follow-up through virtual clinics.

## Introduction

The pandemic of coronavirus disease 2019 (COVID-19) has become the most pressing health concern in the world today [1]. Patients with an autoimmune disease, especially type 1 diabetes (T1D), have a huge risk of getting infected with the virus and becoming severely ill in comparison to normal people since they have a weak immune system [2].

T1D is one of the most common endocrine conditions among children. Globally, the incidence of T1D increases about 3%-4% yearly, with younger age of onset than before. The Kingdom of Saudi Arabia (KSA) has the highest incidence of T1D among the Middle Eastern and North African regions, accounting for one-quarter of the cases [3]. According to a screening project of T1D in KSA that was conducted from 2001 to 2007, the prevalence rate of children and adolescents with T1D is 109.5 per 100,000. Furthermore, it was lowest in the eastern province (48 per 100,000) and highest in the central region (126 per 100,000) [4].

Since the emergence of COVID-19 and a further increase in the spread of the disease, the Saudi ministry of health decided to impose a lockdown to counter the infection and prevent its spread. As a result, the lockdown period caused challenges to the majority of people, especially the diabetic patients, resulting in significant changes in lifestyle, including physical inactivity, unhealthy diet, and psychological distress. Consequently, patients may face difficulties in having access to medications, as well as their follow-up with their physicians.

However, there are limited studies in KSA that measure the impact of the COVID-19 pandemic lockdown on children with T1D. Therefore, this study aims to assess the impact of COVID-19 lockdown on children with T1D in Al-Khobar, KSA, in terms of blood glucose control, medication adherence, development of complications, psychological health, and lifestyle changes.

## Materials and methods

Study design

This is a questionnaire-based cross-sectional study that was conducted between the 30th of October till the 28th of February 2021. 

Study population

Pediatric patients, aged ≤18 years old, with T1D enrolled in the endocrine clinic at King Fahd Hospital of the University (KFHU), in Al-Khobar, KSA. Both genders and all nationalities were included. Patients with type II diabetes, gestational diabetes and maturity-onset diabetes of the young (MODY), permanent neonatal diabetes, and all types of syndromic and monogenic diabetes were excluded from the study. 

Sampling technique and sample size

A simple random sampling method was used with a sample size of 164 children. This study was conducted after the reception of approval from the Institutional Review Board (IRB-2021-098-Med) of Imam Abdulrahman Bin Faisal University (IAU). 

Study variables

Independent variable: COVID-19 lockdown. Dependent variables: impact on follow-ups, blood glucose control, medication adherence, complications, psychological health, and lifestyle changes. Other variables: demographic and clinical characteristics.

Data collection

Tool: Patients were interviewed over the telephone, using an Arabic, anonymous questionnaire. The questionnaire included five parts. The first part covered the socio-demographic information, including age, gender, nationality, height, weight, and the presence of any family history of T1D. The second part comprised general questions about the illness status, including date of diagnosis, type of treatment, way of measuring blood glucose level, and the presence of recent infection with COVID-19. The third part included general information regarding the child status before and during COVID-19 lockdown, such as the compliance of treatment and follow-ups, diabetic complications, and lifestyle patterns. The fourth part emphasized the changes that may occur during the lockdown period such as changes in weight and food consumption. The last part included two questions to assess the psychological status of children with T1D during the lockdown period. Content validity, face validity, and a pilot study were conducted among 10 people who were randomly selected and answered the questionnaire, which was clear for them. Also, the questionnaire was reviewed and finalized by two consultants each from the pediatric and family medicine departments. 

Procedure: A total of 164 patients with their contact information were identified from the database of the endocrine clinic at KFHU and they were interviewed through mobile phone from 30th October to 28th February 2021. The study was ethically conducted, informed consent was obtained before the interview and all participants are allowed to refuse or to voluntarily participate. In addition, participants were informed that all of their information will be secured and for study purposes only. 

Data analysis

The normality test was applied to the variables, the normally distributed data were described using the mean and standard deviation, while the non-normally distributed data were described using the median and interquartile range. Descriptive analysis based on the frequency and percent distribution was done to identify the all-categorical variables including socio-demographic variables of the patients, general data about the illness, compliance of regular follow up with endocrine clinic before the lockdown, general information regarding the access to health care services, dietary change, change in weight and psychological status assessment during the lockdown. Continuous variables including age, weight, height, body mass index (BMI), and hemoglobin A1c (HbA1c) were calculated using a mean score with standard deviation. A comparison between ordinal variables was made by the Wilcoxon signed rank test. All statistical analysis was done using two-tailed tests and a P value less than 0.05 was considered statistically significant. Data were analyzed by using Statistical Package for the Social Sciences (SPSS) version 24 (IBM Corp., Armonk, NY).

## Results

Characteristics of the participants 

**Figure 1:**
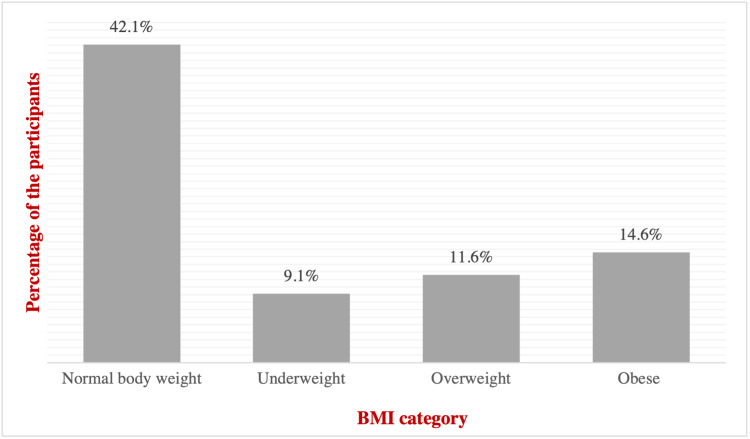
Body mass index (BMI) of the participants (n=146)

The study included 164 children with T1D. The majority were Saudis (94.5%) and females (54.9%). The mean age of the children was (12.45 ± 3.66) years old, and the mean weight and height were (41.66 ± 1.26) kg and (141.51 ± 2.10) cm, respectively (Table 1). Most of the patients had normal BMI (45.1%), while 14.6% of them were obese. The mean BMI was (21.49 ± 1.90) (Figure 1).

**Table 1:** Demographic characteristics of the participants (n=164)

Variables		n	Percent (%)
Nationality	Saudi	155	94.5*
	Non-Saudi	9	5.5
Gender	Male	74	45.1
	Female	90	54.9*
BMI	Normal body weight	69	42.1*
	Underweight	15	9.1
	Overweight	19	11.6
	Obese	24	14.6
*indicates the highest percent			

General data about the illness 

The majority of the children (73.8%) were receiving two and more of long-acting insulin, intermediate-acting insulin, short-acting insulin, and premixed insulin. Moreover, 47% of them were taking their medications with their parent’s assistance. About 70.1% of them were taking the medication 3-4 times daily, and only 12.2% reported using an insulin pump. Regarding blood glucose monitoring, 40% of the patients were measuring it by themselves, while 50% of them were using the continuous glucose monitoring device.

Fortunately, 93.9% of the patients did not get infected with COVID-19, and only 6.1% were infected. Furthermore, 11.6% of the patients reported having other comorbidities such as allergies, thyroid diseases, asthma, G6PD deficiency, and growth hormone deficiency (Table 2).

**Table 2:** General data about the illness (n=164)

Variables		Frequency	Percent (%)
Type of Medication Used^‡^	Long acting Insulin	9	5.5
	Intermediate acting Insulin	1	0.6
	Short acting Insulin	17	10.4
	Premixed insulin	7	4.3
	Two and more of the above	121	73.8*
	Don't know	9	5.5
The Frequency of Medication Use Daily	Doesn’t take the medication	2	1.2
	1-2 times	11	6.7
	3-4 times	115	70.1*
	5-6 times	17	10.4
	Other	19	11.6
The Use of Insulin Pump	Yes	20	12.2
	No	144	87.8*
Administration of The Medication	Parents	77	47.0*
	Self	40	24.4
	Both	47	28.7
The Type of Blood Glucose Monitor Device	Self-monitoring blood glucose	66	40.2
	Continuous glucose monitor	83	50.6*
	Both	11	6.7
	Other	4	2.4
COVID-19 Infection	Yes	10	6.1
	No	154	93.9*
Other Comorbidities	Allergic diseases	4	2.4
	Asthma	3	1.8
	Thyroid diseases	4	2.4
	Other (G6PD deficiency, growth hormone deficiency)	7	4.3*
*indicates the highest percent			
^‡^More than one answer			

The compliance with regular follow up before the lockdown 

**Figure 2:**
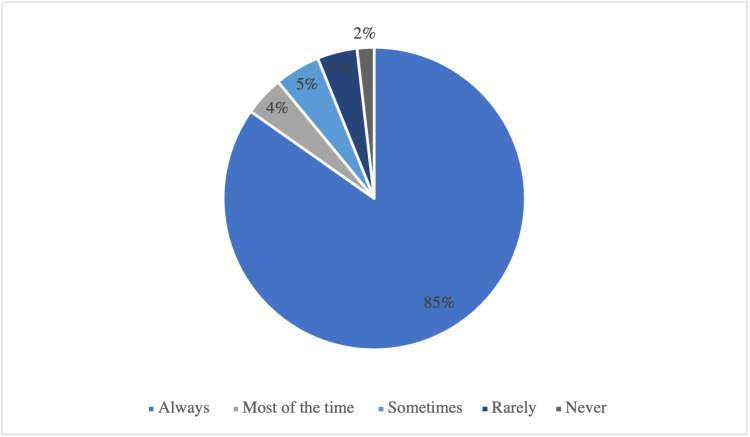
The compliance with regular follow-up with the endocrine clinic before the lockdown (n=164)

84.76% of the children reported well compliance to regular follow-up with the endocrine clinic before the lockdown, while only 1.83% reported that they have never followed up regularly (Table 3) (Figure 2).

**Table 3:** The compliance with regular follow-up with the endocrine clinic before the lockdown (n=164)

Compliance with Regular Follow-up with The Doctor Before the Lockdown	n	Percent (%)
Always	139	84.76*
Most of the time	7	4.26
Sometimes	8	4.88
Rarely	7	4.27
Never	3	1.83
*indicates the highest percent		

Comparison of general child status before and during COVID-19 lockdown 

The comparison of general child status before and during COVID-19 lockdown was done using the Wilcoxon signed-rank test. A statistically significant difference was found for four questions, and no significant difference was found for the other six questions. While comparing the patient’s compliance on medication, 71.3% of them were taking their medications regularly before the lockdown. This percentage raised slightly during the lockdown to 72.6%. However, that was not statistically significant (P=0.434). On the other hand, the percentage of children who did not miss any dose of medications before and during lockdown has significantly increased from 70.7% to 92.7%, which was statistically significant (P=0.000).

Moreover, there was no statistically significant difference in the frequency of measuring blood glucose level, the number of hypo/hyperglycemic attacks, and the need for an insulin corrector dose due to hyperglycemia before and during the lockdown. Also, the need for visiting the emergency department due to hypoglycemia, and the need for ICU admission due to DKA had significantly decreased during the lockdown (P=0.001). In addition, the percentage of patients who are not calculating the carbohydrate contents of the food during the lockdown increased to 61.6% in comparison to 56.7% before the lockdown, however, it was not statistically significant (P=0.320). More than half of the patients (68.3%) tend to consume three meals per day during the lockdown while only 53.7% of them used to do that before the lockdown (P=0.005) (Table 4).

**Table 4:** Comparison of general child status before and during COVID-19 lockdown  (n=164) DKA: diabetic ketoacidosis, ER: emergecy room, ICU: intensive care unit.

Questions	Before COVID-19 Lockdown (n=164)		During COVID-19 Lockdown (n=164)		P. value
Using the Medication Regularly	n	(%)	n	(%)	0.434
Always	117	71.3 *	119	72.6 *	
Most of the time	20	12.2	24	14.6	
Sometimes	20	12.2	14	8.5	
Rarely	2	1.2	4	2.4	
Never	5	3.0	3	1.8	
Missing Any Dose of The Medication	n	(%)	n	(%)	0.000
More than 6 times a week	7	4.3	12	7.3	
5-4 times a week	7	4.3	0	0.0	
3-2 times a week	22	13.4	0	0.0	
Once weekly	12	7.3	0	0.0	
Never	116	70.7 *	152	92.7 *	
The Frequency of Measuring Blood Glucose Daily	n	(%)	n	(%)	0.062
More than 6 times daily	59	36.0 *	58	35.4 *	
4-6 times daily	39	23.8	24	14.6	
1-3 times daily	53	32.3	38	23.2	
Never	13	7.9	44	26.8	
The Frequency of Experiencing of Hyperglycemic Symptoms Weekly	n	(%)	n	(%)	0.073
More than 6 times a week	33	20.1	39	23.8	
5-4 times a week	19	11.6	23	14.0	
3-2 times a week	29	17.7	22	13.4	
Once weekly	26	15.9	29	17.7	
Never	57	34.8 *	51	31.1 *	
The Need of a Corrector Insulin Dose as a Result Of Hyperglycemia	n	(%)	n	(%)	0.336
More than 6 times a month	58	35.4*	58	35.4*	
5-4 times a month	24	14.6	18	11.0	
3-2 times a month	17	10.4	18	11.0	
Once month	14	8.5	23	14.0	
Never	51	31.1	47	28.7	
The Frequency of Experiencing Hypoglycemic Symptoms Weekly	n	(%)	n	(%)	0.066
More than 6 times a week	6	3.7	5	3.0	
5-4 times a week	11	6.7	6	3.7	
3-2 times a week	42	25.6	32	19.5	
Once weekly	33	20.7	32	19.5	
Never	71	43.3 *	89	54.3 *	
The Frequency of ER Visits as a Result of Hypoglycemia	n	(%)	n	(%)	0.002
More than 6 times a month	14	8.5	4	2.4	
5-4 times a month	3	1.8	16	9.8	
3-2 times a month	6	3.7	4	2.4	
Once month	16	9.8	1	0.6	
Never	125	76.2 *	139	84.8 *	
The Frequency of ICU Admission as a Result of DKA	n	(%)	n	(%)	0.001
More than 6 times a month	1	0.6	0	0.0	
5-4 times a month	0	0.0	0	0.0	
3-2 times a month	1	0.6	0	0.0	
Once month	6	3.6	7	4.3	
Never	127	77.4 *	148	90.2 *	
Other	29	17.7	9	5.5	
Number of Meals Consumed Per Day	n	(%)	n	(%)	0.005
More than 4	64	39.0	45	27.4	
3 time a day	88	53.7 *	112	68.3 *	
2 times a day	9	5.5	5	3.0	
One time a day	3	1.8	2	1.2	
Calculation of The Carbohydrate Contents of The Food and The Insulin Doses Before Meals	n	(%)	n	(%)	0.320
Yes	63	38.4	60	36.6	
No	93	56.7 *	101	61.6 *	
Sometimes	8	4.9	3	1.8	
*indicates the highest percent					

**Figure 3:**
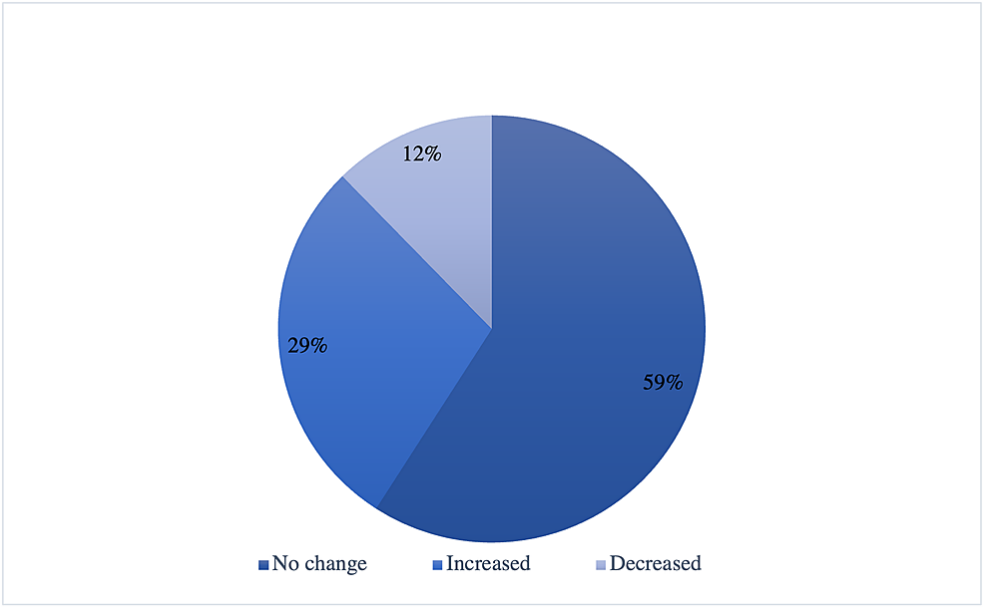
Change in the amount of food (n=164)

**Figure 4:**
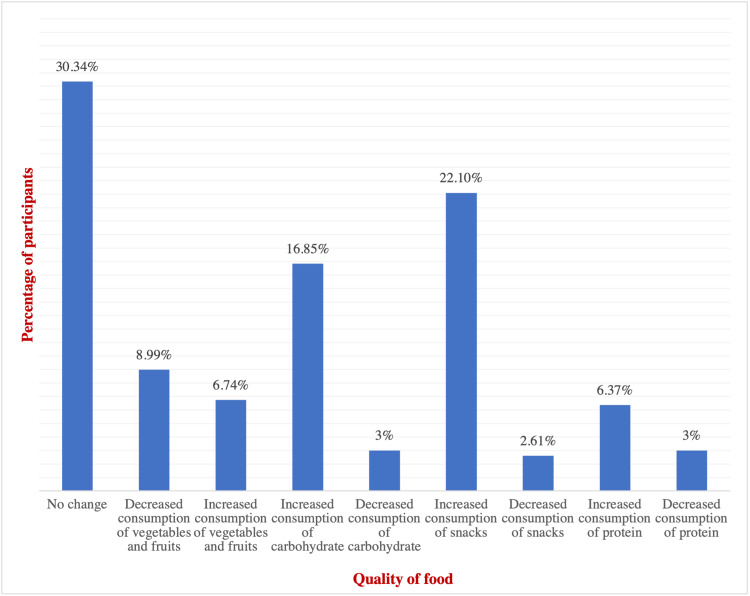
Change in the quality of food during the lockdown (n=164)

**Figure 5:**
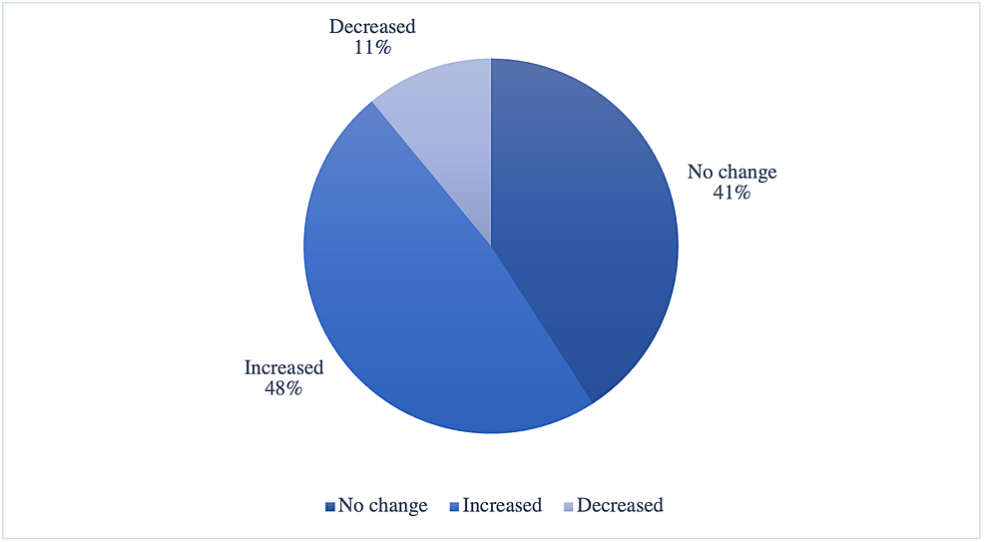
Change in weight during the lockdown (n=164)

The assessment of food consumption during the lockdown revealed that almost half of the children (59.1%) reported no change in the amount of food consumption (Table 5) (Figure 3). Concerning the quality of food, 30%-34% reported no change, while 22.1% of them reported an increase in snacks consumption (Table 5) (Figure 4). Regarding weight change during the lockdown, the majority of patients (48.17%) reported an increase in their weight, whereas 40.85% reported no change in their weight, and only 10.98% experienced weight loss (Table 6) (Figure 5).

**Table 5:** General information regarding the diet during COVID-19 lockdown (n=164)

Variable	Frequency	Percent (%)
Change in The Amount of Food		
No change	91	59.1*
Increased	44	28.58
Decreased	19	12.32
Change in The Quality of Food^‡^		
No change	81	30.34*
Decreased consumption of vegetables and fruits	24	8.99
Increased consumption of vegetables and fruits	18	6.74
Increased consumption of refined sugars	45	16.85
Decreased consumption of refined sugars	8	3
Increased consumption of snacks	59	22.1
Decreased consumption of snacks	7	2.61
Increased consumption of protein	17	6.37
Decreased consumption of protein	8	3
*indicates the highest percent		
^‡^More than one answer		

**Table 6:** Change in weight during the lockdown (n=164)

Change in the weight during the lockdown	n	Percent (%)
No change	67	40.85
Increased	79	48.17*
Decreased	18	10.98
*indicates the highest percent		

Comparison between HbA1C level before and during lockdown 

The mean of children's HbA1C before the lockdown was (9.85± 0.80), and during the lockdown (9.78±1.03). That indicates there was no difference in the level of HbA1C before and during the lockdown (Table 7).

**Table 7:** Comparison between HbA1C level before and during lockdown HbA1C: hemoglobin A1c.

HBA1C	Mean	Std. Deviation
Last HbA1C reading before the lockdown	9.85	0.801
Last HbA1C reading during the lockdown	9.78	1.03

Access to health care services during COVID-19 lockdown 

**Figure 6:**
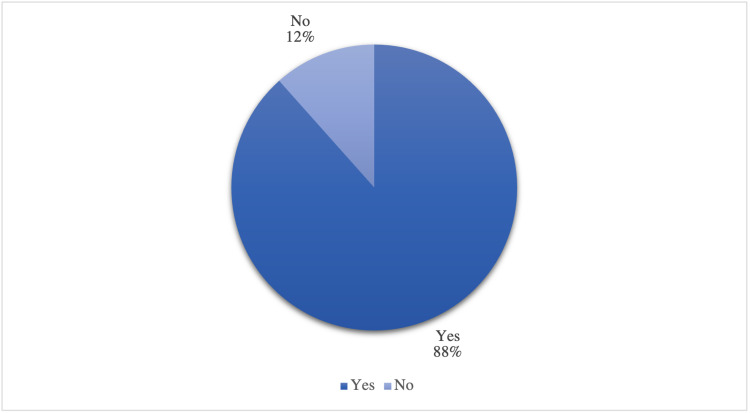
The access to all medical needs such as insulin injections and insulin pump during the lockdown (n=164)

**Figure 7:**
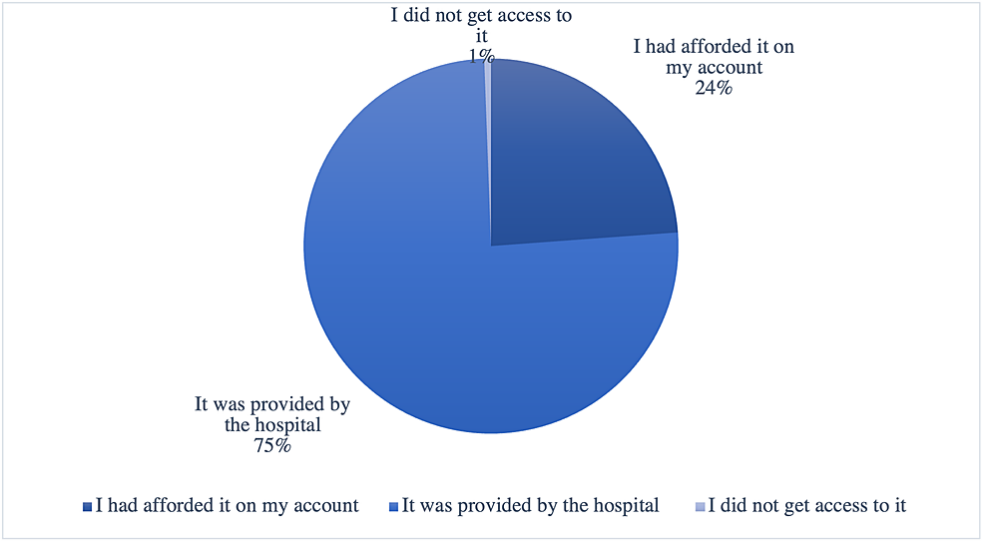
The way of providing medical needs (n=164)

Most of the patients (88.41%) had access to all medical needs as insulin injections and pumps during the lockdown, while a small percentage (11.59%) had no access (Table 8) (Figure 6). 75.58% of patients were supplied with medical needs by the hospital, while 23.84% bought it themselves, and less than 1% of patients did not get access to it (Table 8) (Figure 7).

**Table 8:** General information regarding the access to health care services during COVID-19 lockdown (n=164)

Variable	n	Percent (%)
Access to all medical needs such as insulin injections and insulin pump during the lockdown		
Yes	145	88.41*
No	19	11.59
	Frequency	Percent (%)
Method of communication with the doctor during the lockdown^‡^		
Using virtual clinic	16	8.84
Using mobile calls	74	40.33*
Visiting another doctor (like private hospitals)	14	7.73
Keep visiting the same doctor at the clinic	15	8.29
No communications	62	34.81
The way of Providing medical needs, such as insulin and insulin pumps^‡^		
I had afforded it on my account	41	23.84
It was provided by the hospital	130	75.58*
I did not get access to it	1	0.58
*indicates the highest percent		
^‡^More than one answer		

**Figure 8:**
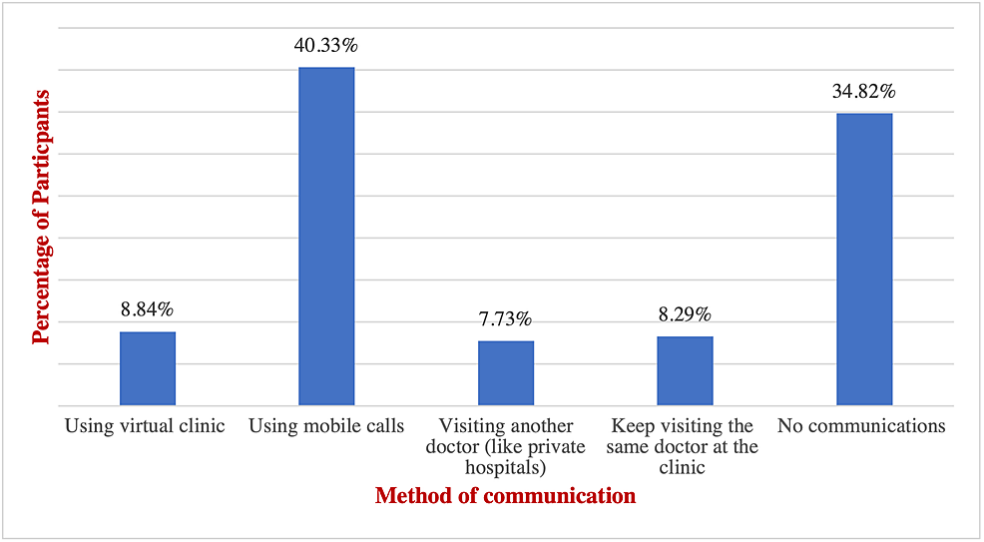
Method of communication with the doctor during the lockdown

With respect to patient-doctor communication and follow-ups during the lockdown, 40.33% of the patients were in contact with their physicians through using mobile phones, 34.81% lost communication completely, 8.84% were using virtual clinics, 8.29% were visiting the same physicians and 7.73% switched to seek another medical advice from different physicians at private hospitals (Figure 8).

Prevalence of mood changes among children with T1D during the lockdown 

For the psychological status assessment, since the majority of children (47%) have never experienced any symptoms suggestive of depression or change in mood during the lockdown, COVID-19 lockdown did not have a negative effect on TID children's psychological status (P=0.123) (Table 9).

**Table 9:** Psychological status assessment during the lockdown (PHQ2)

Items	Never		Sometimes		Often		Always		P.value
	F	%	F	%	F	%	F	%	
During the lockdown My child is no longer enjoying his usual entertainment activities	77	47.0*	39	23.8	23	14.0	25	15.2	0.123
During the lockdown My child seems depressed and had low mood most of the time	77	47.0*	49	29.9	27	16.5	11	6.7	
*indicates the highest percent									

## Discussion

Diabetes mellitus is a major autoimmune disease that is associated with multiple complications [5]. Having uncontrolled blood glucose levels can significantly increase the incidence of complications and mortality rates [6]. Maintaining good glycemic control, complying with medications, and adopting a healthy lifestyle is essential to prevent these complications [7]. Therefore, we want to clarify whether COVID-19 lockdown will impose a significant impact on T1D patients.

In this study, few children were infected with COVID-19 (6.1%,), due to the extensive precautionary measures implemented by the government and the children's families. However, many cases may be underdiagnosed due to fear of being infected and isolated from their families. Another reason for underdiagnosis is getting confused between the common cold and COVID-19 symptoms. d'Annunzio G et al. also stated that there was no association between T1D and increased risk of COVID-19 infection [6]. 

Regarding glycemic control, there were no differences in the HbA1C levels before and during the lockdown, and the glycemic control was not affected significantly by the lockdown. This is probably is due to recall bias as many children or their families do not recall the HbA1C results during and after the lockdown. In contrast, Verma A et al. reported an increase in the HbA1C level during the lockdown compared to before which was (9.78±1.03) and (9.85± 0.80) respectively [1]. In another study done in Italy, which examined the blood glucose level before and during the lockdown on T1D patients, they found an improvement in the glycemic control during the lockdown in comparison to before it [7].

Concerning the medication compliance among children with T1D, it was tested before and during the lockdown and the results showed that the number of children who were always taking their medication regularly has increased from 71.3% to 72.6% with the percentage of not missing any doses increased from 70.7% to 92.7%, (P=0.000) (before and during lockdown respectively). The reason behind that is, parents were more capable of taking care of diabetic children as a result of working from home during the lockdown. On the other hand, a cross-sectional study was done in India that included 52 children with T1D measured the level of medication compliance before and during the lockdown and it revealed a significant reduction in the level of medication compliance during the lockdown. Therefore, glycemic control was worsened during lockdown [1].

The dietary intake improved during the lockdown in our study, which the percentage of children who were consuming three meals per day has increased from 53.7% before the lockdown up to 68.3% during the lockdown period, which was statistically significant (P=0.005). That is probably due to the good diet scheduling with their family during the lockdown. In addition, decreased meetings with non-diabetic peers affected them positively regarding the intake of high sugar beverages and desserts. Consequently, emergency room visits as a result of hypoglycemia have decreased from 23.8% before the lockdown to 15.2% during the lockdown, which was statistically significant (P= 0.002). In contrast, Passanisi S et al. stated that more than half of the patients (56.9%, did not report any difference concerning dietary intake between the two periods [8]. Moreover, Verma A et al. stated that (82.6%, n=42) had no dietary habit changes during the lockdown period compared to before [1,8].

In this current study, about 45.7% of children during the lockdown reported having more than one episode of hypoglycemia weekly compared to 56.7% before the lockdown and that was non statistically significant (P=0.066). Moreover, 68.9% of the children experienced more than one episode of hyperglycemic symptoms weekly during the lockdown compared to 65.2% before the lockdown. Only 71% out of them needed a corrector insulin dose monthly during the lockdown in comparison to before the lockdown 68.9%. Moreover, only seven children had ICU admissions during the lockdown as a result of DKA. The frequency of ICU admission during the lockdown has decreased as compared to before the lockdown (4.8% and 4.3%, respectively). Verma A et al. have reported similar results in which the number of patients who experienced hyperglycemia, hypoglycemia, and hospitalization as a result of DKA during the lockdown was 36.5%, 15.3%, and 7.7%, respectively [8].

Surprisingly, 47% of the children were enjoying their usual entertainment activities and did not seem depressed or in a low mood during the lockdown period. COVID-19 pandemic and lockdown have only a minimal effect on T1D patients (6.7%) who reported depression and low mood most of the time. COVID-19 lockdown did not have a significant psychological impact on children with T1D. Results of this study were in concordance with the study which was conducted in India to measure the impact of COVID-19 lockdown upon patients with T1D [1]. On the other hand, those results were contrary to the study that was done in Germany, which showed a significant increase in the emergence of new cases of T1D in which may be attributed to an increase in psychological stress during a stressful catastrophic event in such a COVID-19 pandemic [9].

## Conclusions

This study aims to assess the impact of the COVID-19 lockdown on pediatric T1D patients in Al-Khobar, KSA. The results indicate that the percentage of compliance to medications in the period during the lockdown was significantly higher than the percentage before the lockdown (92.7% vs 70.7%, respectively). No statistically significant difference was found in the frequency of measuring blood glucose level, the number of hypo/hyperglycemic attacks, and the need for an insulin corrector dose before and during the lockdown. Moreover, the need for emergency department visits due to hypoglycemia and ICU admission due to DKA has been significantly decreased during the lockdown. This study has clearly demonstrated a negative impact of the pandemic on glycemic control in T1D, a chronic condition that needs regular attention. The factors responsible such as non-availability of insulin/glucostrips, poor dietary compliance and decreased physical activity need to be taken into consideration in planning and addressing chronic health conditions in future pandemics because if appropriate action is not taken, there would be a considerable increase in health care costs. The results of the study will be beneficial to encourage medical practitioners to educate and raise awareness of the importance of self-blood glucose monitoring, healthy lifestyle, psychological wellbeing, and regular follow-up through virtual clinics in order to support diabetic patients and prevent the development of further complications.
